# Chitosan-Based Delivery of Avian Reovirus Fusogenic Protein p10 Gene: *In Vitro* and *In Vivo* Studies towards a New Vaccine against Melanoma

**DOI:** 10.1155/2020/4045760

**Published:** 2020-06-13

**Authors:** Claudia Robles-Planells, Carlos Barrera-Avalos, Leonel E. Rojo, Eugenio Spencer, Marcelo Cortez-San Martin, Silvia Matiacevich, Jorge Pavez, Luis A. Milla, Franco D. Navarro, Brandon A. Martínez, Francisco J. Bravo, Andrea Mella, Juan Pablo Huidobro-Toro, Ricardo Fernandez, Alejandro Escobar, Claudio Acuña-Castillo

**Affiliations:** ^1^Departamento de Biología, Facultad de Química y Biología, Universidad de Santiago de Chile, USACH, Alameda, 3363 Santiago, Chile; ^2^Centro de Biotecnología Acuícola, Universidad de Santiago de Chile, USACH, Alameda, 3363 Santiago, Chile; ^3^Centro de Nanociencias y Nanotecnología, Universidad de Santiago de Chile, USACH, Chile; ^4^Centro de Tecnología de los Alimentos, Facultad Tecnológica, Universidad de Santiago de Chile, USACH, Alameda, 3363 Santiago, Chile; ^5^Departamento de Química de los Materiales, Facultad de Química y Biología, Universidad de Santiago de Chile, USACH, Alameda, 3363 Santiago, Chile; ^6^Centro de Investigación Biomédica y Aplicada (CIBAP), Escuela de Medicina, Facultad de Ciencias Médicas, Universidad de Santiago de Chile, Chile; ^7^Departamento de Salud, Universidad de Los Lagos, Osorno, Chile; ^8^Laboratorio Biología Celular y Molecular, Instituto de Investigación en Ciencias Odontológicas, Facultad de Odontología, Universidad de Chile, Santiago, Chile

## Abstract

Reovirus is known to have an anticancer effect in both the preclinical and clinical assays. Current evidence suggests that the reovirus-mediated impact on tumor growth depends on the activation of specific antitumor immune responses. A feasible explanation for the oncolytic effects and immune system activation is through the expression of the fusogenic reovirus protein. In this work, we evaluated the *in vivo* antitumor effects of the expression of fusogenic protein p10 of avian reovirus (ARV-p10). We used chitosan nanoparticles (CH-NPs) as a vehicle for the ARV-p10 DNA in murine B16 melanoma models both *in vitro* and *in vivo*. We confirmed that ARV-p10 delivery through a chitosan-based formulation (ARV-p10 CH-NPs) was capable of inducing cell fusion in cultured melanoma cells, showing a mild cytotoxic effect. Interestingly, intratumor injection of ARV-p10 CH-NPs delayed tumor growth, without changing lymphoid populations in the tumor tissue and spleen. The injection of chitosan nanoparticles (CH-NPs) also delayed tumor growth, suggesting the nanoparticle itself would attack tumor cells. In conclusion, we proved that *in vitro* ARV-p10 protein expression using CH-NPs in murine melanoma cells induces a cytotoxic effect associated with its cell fusion. Further studies are necessary for establishing a protocol for efficient *in vivo* DNA delivery of fusion proteins to produce an antitumoral effect.

## 1. Introduction

Virotherapy is an alternative therapy against cancer, which takes advantage of the cytolytic activity of viruses during their infective cycle and the absence of response mechanisms of the tumor cells against viruses. Fusogenic oncolytic viruses (FOVs) show some advantages over nonfusogenic viruses when used against cancer cells, mainly because FOVs can induce tumor immunogenic cell death (ICD), producing cellular structures with strong immune-stimulatory effects [1]. The first virus used against cancer was a modified herpes simplex virus. It was aimed at obtaining efficient and safe therapy against unresectable stages of melanoma. This therapy was approved in 2015 by the FDA. Since then, other therapies based on oncolytic viruses alone or in combination with other treatments are being researched [2]. Moreover, some viruses have demonstrated promising results in clinical trials [3, 4]. The mode of action of these therapies is associated with efficient malignant cell death, mediated by the direct viral cytotoxic effect and/or stimulation of the immune system [5, 6].

Reovirus (RV) is a double-stranded RNA (dsRNA) virus without a membranous envelope expressing a nonstructural small fusion-associated membrane protein (FAST protein (Fusion-Associated Small Transmembrane protein)) in an active conformation in the cell membrane of infected cells [7]. This protein expressed at late stages for the viral cycle leads to syncytium formation, a mechanism involved in the horizontal propagation of the viral infection [8, 9]. RV also displays tropism and efficiently replicates in tumor cells with the activated Ras pathway [10]. These characteristics allow the use of RV in oncological therapy, either alone or combined with the conventional and nonconventional treatments [11]. For instance, a combination of RV and cisplatin enhanced cytotoxicity in the human and murine melanoma cell lines *in vitro* and murine tumors *in vivo* synergistically [12]. Intratumor (i.t.) reovirus injection, together with intravenous (i.v.) anti-PD-1 antibody, significantly enhanced survival of mice with subcutaneous B16 melanoma. In both cases, the mechanism is dependent on the activation of antitumor immune responses [13]. Currently, RV is used in cancer therapeutics under the name REOLYSIN®. This product corresponds to a formulation of the human reovirus (HRV), tested at the preclinical stage and Phase I, Phase II, and Phase III clinical studies in a broad range of cancer indications [11]. For example, REOLYSIN® combined with carboplatin and paclitaxel is a safe and potentially efficacious therapy for patients with advanced malignant melanoma [14]. Evidence suggests that the antitumoral mechanism associated with RV involves the activation of the immune response related to fusogenic activity and ICD induction. These effects have only been reported for reovirus FAST expression. Le Boeuf and coworkers, using an interferon-sensitive vesicular stomatitis virus (mutant VSV*Δ*M51) encoding the fusogenic p14 FAST protein, showed an increased anticancer effect in two different *in vitro* cancer systems (MCF-7 and 4T1). This strategy also produced positive results *in vivo*, extending the survival of animals in 4T1 and CT26 metastatic colon cancer, with a mechanism associated with the activation of antitumor immune responses [15]. In this study, we evaluated the effect of *in situ* transfection of the avian RV (ARV) FAST protein, named p10, on murine B16 melanoma tumor growth and induction of an immune response using chitosan nanoparticles (CH-NPs) as a vehicle to deliver DNA into cancer cells.

## 2. Materials and Methods

### 2.1. Nanoparticle Generation and Characterization

The gene coding the p10 protein of avian reovirus (ARV-p10) inserted into the vector pUC57 was subcloned into the commercial expression vector for eukaryotic cells pIRES2 (BD Biosciences Clontech, PT3267-5) using the same strategy that we described previously [16]. Complexes were generated by the coacervation method and characterized as we previously described using chitosan at an N/P 20 ratio, chosen due to its homogeneity and transfection efficiency.

Transfection efficiency was verified in B16 cells using a green fluorescence protein expression vector as a reporter (pcDNA3.1-GFP), determined by the percentage of GFP-positive cells (GFP+) relative to untreated cells 48 hours posttreatment. Fluorescence was monitored by flow cytometry using BD Accuri C6 equipment (BD Biosciences, USA). Lipofectamine 2000 (Invitrogen, 11668027) was used as a positive transfection control, and naked pcDNA3.1-GFP was used as a negative control.

### 2.2. ARV-p10 Transcript Expression

The expression of ARV-p10 mRNA in B16 cells transfected with nanoparticles at N/P 20 was evaluated by conventional RT-PCR (Fw 5′-CAGGGTCATGTAACGGAGCTA-3′ and Rv 5′-CAGCAGGAATCCTCCTCCAGC-3′) 48 hours posttransfection, using enzyme glyceraldehyde 3-phosphate dehydrogenase (GAPDH) transcript as a constitutive expression control (Fw 5′-TCGGTGTGAACGGATTTGGC-3′ and Rv 5′-TTTGCCGTGAGTGGAGTCATACTG-3′). Briefly, the cells were harvested, and the total RNA was isolated with TRIzol® Reagent (Gibco, 15596026) according to the manufacturer's recommendations. Subsequently, 1 *μ*g of total RNA was treated with DNAse (RQ1 DNAse-free RNAse, Promega, M610A) for 30 min at 37°C and then used for cDNA synthesis using reverse transcriptase M-MLV and OligodT15 (Promega, C1101) according to the manufacturer's instructions. After PCR, the DNA product was observed on a 1% agarose gel staining with GelRed (Biotium, 41002).

### 2.3. ARV-p10 Protein Expression

For ARV-p10 detection, B16 cells at a confluence of 40-60% were transfected with NPs of chitosan and pIRES-ARV (N/P 20) and Lipofectamine (Invitrogen, 11668027). At 48 hours posttransfection, the cells were washed with PBS, fixed with 4% paraformaldehyde for 10 min, washed again, and blocked with goat serum at 10% in PBS for 1 hour at room temperature. Subsequently, they were incubated with a rabbit anti-p10 polyclonal serum (gently donated by Dr. Roy Duncan, Dalhousie University, Nova Scotia) at a dilution of 1 : 500 in 10% goat serum at 4°C overnight with gentle agitation. Cells were washed for 10 min with PBS three times in gentle agitation and incubated in the dark for 1 hour at room temperature with an anti-rabbit IgG monkey secondary antibody conjugated with Alexa Fluor 546 (LifeTech, A10040) at a dilution of 1 : 2000 in 10% goat serum. A new cycle of washes was repeated before incubating for 5 min with DAPI 0.5 mg/ml. Finally, coverslips were mounted on slides using ProLong Gold (Invitrogen, P36930). Samples were visualized in the Zeiss LSM 800 confocal microscope (Universidad de Santiago de Chile).

### 2.4. Cell Fusion

Cell fusion was further confirmed by evaluating the presence of syncytium in transfected B16 cells. Briefly, B16 cells seeded on coverslips at a 40-60% of confluence were transfected with 0.5 *μ*g of the pIRES-ARV plasmid using Lipofectamine 2000 (Invitrogen, 11668027) according to the manufacturer's recommendations and 2.5 *μ*g of the same plasmid present in chitosan NPs synthesized at an N/P 20. At 48 hours posttransfection, the cells were washed with PBS and incubated with the CellMask Orange plasma membrane stain probe (Life Technologies, C10045) according to the manufacturer's recommendations. After fixation with 3.75% paraformaldehyde for 30 min at 37°C, the cells were incubated with DAPI 0.5 mg/ml for 5 min. Covers were mounted on slides with DABCO and observed using the Zeiss LSM 800 confocal microscope (Universidad de Santiago de Chile). For hemacolor stain, cells were washed and fixed with at 3 : 1 solution of methanol : acetic acid for 15 min and stained with hemacolor stain (Sigma-Aldrich) according to the manufacturer. Syncytia were counted in 5 random planes using a conventional inverted microscope.

### 2.5. Cytotoxicity

The effect on posttransfection cell viability was evaluated by the determination of metabolic activity in transfected B16 cells. Briefly, 3.5 × 10^4^ B16 cells were transfected with chitosan nanoparticles or Lipofectamine containing 2.5 *μ*g of plasmids. 24, 48, and 120 hours posttransfection, MTT (Sigma-Aldrich, M2128) assays were performed according to the manufacturer's suggestions. The absorbance value at 570 nm of untransfected cells was used as a 100% viability.

### 2.6. Antitumor Treatment

Mice of 8 to 10 weeks of strain C57BL/6J were obtained from the animal facility of Facultad de Química y Biología from the Universidad de Santiago de Chile. Animals were maintained with *ad libitum* feeding under a light/dark cycle. All protocols were approved by the Bioethics Committee of Universidad de Santiago de Chile (Letter No. 489).

B16 cell suspensions of 2 × 10^5^ living cells were used to induce tumor development in C57BL/6J mice by subcutaneous (s.c.) injection into the lumbar region (challenge), as described previously [17]. Once a tumor reached a volume of 2.0 mm^3^, animals were separated into three groups: (i) without treatment, (ii) CH: treated with chitosan, and (iii) NP-ARV: treated with NPs of chitosan+pIRES-ARV. The treatment with NP-ARV consisted of an intratumor injection of an NP suspension synthesized at an N/P 20, composed by 122 *μ*g of chitosan and 10 *μ*g of plasmid pIRES-ARV, in 100 *μ*l of PBS. The same amount of chitosan was used to treat the CH group of mice. The tumor growth was evaluated by measuring the tumor size using a caliper and calculating tumor volume according to the half-sphere formula (*V* = 2/*πr*^3^, where *V* corresponds to volume in mm^3^ and *r* to tumor radius in mm). A maximum tumor volume (MTV) of 260 mm^3^ was used as the endpoint criterion, at which time the animals were sacrificed by cervical dislocation and processed for subsequent analyses.

### 2.7. Splenocytes and Tumor-Infiltrating Lymphocytes

The spleen and tumor of each animal were removed. The spleen was disgregated in a 100-mesh metal grid and then treated with ACK buffer (155 mM NH_4_Cl, 10 mM KHCO_3_, and 1 mM Na_2_EDTA, pH 7.3) for 5 min with gentle shaking to remove, by differential lysis, erythrocytes. After centrifugation for 7 min at 600 g, the splenocytes were resuspended in RPMI 1640 medium with 10% FBS (Biological, DW105804-127-1A).

In parallel, the tumor was removed and received on a plate with 5% FBS (Biological, DW105804-127-1A) in HBSS buffer (Gibco, 24020117) on ice and disintegrated using scissors. The homogenate was collected and treated with 1 mg/ml type IV collagenase (Sigma, C-5138) and 0.05 mg/ml DNAse (Promega, M6101) for 30 minutes at 37°C with gentle agitation. The digested extract was screened using a 100-mesh, and the filtrate was washed with HBSS 5% FBS and centrifuged at 600 g for 7 minutes at 4°C. The cell pellet obtained was treated with ACK erythrocyte lysis buffer (155 mM NH_4_Cl, 10 mM KHCO_3_, and 1 mM Na_2_EDTA, pH 7.3) for 5 min at room temperature. The washing and spinning steps were repeated, the supernatant was removed, and the pellet was resuspended in 40% Percoll (GE Healthcare, 17-0891-01) in HBSS. The same volume of Percoll at 70% was added under the cells-40% Percoll suspension using a glass Pasteur pipette. It was centrifuged at 750 g for 20 min at room temperature with low acceleration and deceleration. Then, the T lymphocytes present between the two phases formed after centrifugation were removed and washed with HBSS 5% FBS at 600 g for 7 minutes at 4°C. Finally, tumor-infiltrating lymphocytes (TIL) were resuspended in RPMI 1640 medium with 10% FBS (Biological, DW105804-127-1A).

### 2.8. T Lymphocyte Activation

For the detection of the intracellular IFN*γ* and IL-17A cytokines, specific of the Th1 and Th17 subpopulations, respectively, 2 × 10^6^ splenocytes and TIL isolated were activated with 0.25 *μ*M of 20 PMA (Sigma, P1585) and 1 *μ*g/ml of Ionomycin (StemCell, 73722). Simultaneously, the vesicular transit was blocked to avoid the release of the cytokines to the extracellular medium, using 10 *μ*g/ml of Brefeldin A (StemCell, 73012). Both treatments were performed for 4 hours at 37°C. with 5% CO_2_. As a negative activation control, cells treated only with Brefeldin A (Nuñez, Saez et al. 2013) were used.

### 2.9. Flow Cytometry Staining

For the detection of lymphocytes in the spleen and TIL, 2 × 10^6^ cells were used. Labeling was performed for 30 min at 4°C in the dark using the antibodies in a 1 : 10 dilution in staining buffer (2% FBS in PBS) for surface antibodies and Fix-Perm buffer (intracellular fixation permeabilization buffer set, eBioscience, 88-8824-00) for intracellular antibodies. To ensure lymphocyte population analysis, the CD45 label was made with the CD45.2-APC anti-mouse antibody (eBioscience, Clone: 104). For the detection of the CD8^+^ and CD4^+^ populations, the anti-mouse CD8a-PE (eBioscience, Clone: 53-6.7) and anti-mouse CD4-FITC (eBioscience, Clone: RM4-5) antibodies were used, respectively. For the detection of CD4^+^ subpopulations, the cells were fixed and permeabilized with Fix-Perm buffer. The CD4^+^ Foxp3^+^ (Treg) population was detected using the anti-mouse/rat Foxp3-PE-Cy5 antibody (eBioscience, Clone: FJK-16s). The CD4^+^ IFN*γ*^+^ (Th1) and CD4^+^ IL-17A^+^ (Th17) populations were detected using the IFN*γ*-PE anti-mouse (eBioscience, Clone: XMG1.2) and IL-17A-PerCP anti-mouse (eBioscience, Clone: TC11-18H10.1) antibodies, respectively, in lymphocytes previously activated with PMA and Ionomycin. BD Accuri C6 equipment (BD Biosciences, USA) was used for the acquisition of flow cytometry data and FlowJo 7.6.1 software (for the population's analysis).

### 2.10. Statistical Analysis

Results were graphed as average value ± SEM and analyzed by the nonparametric Mann-Whitney test. The effect on tumor growth was analyzed by Fischer's exact test. A confidence value of 95% was used. All analyses were performed with the GraphPad Prism 5.01 computer program (GraphPad Software, Inc., USA).

## 3. Results

### 3.1. Nanoparticle Characterization

**Figure 1:**
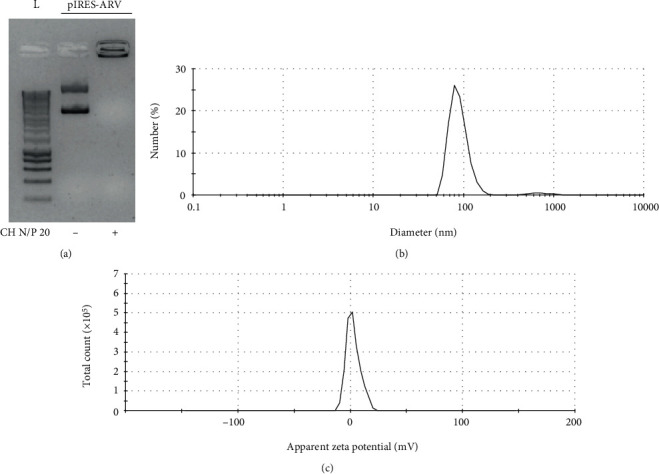
Electrophoretic and physicochemical characterization of chitosan nanoparticles containing pIRES-ARV (NP-ARV). (a) Electrophoretic migration of pIRES-ARV on an agarose gel, (b) size distribution, and (c) surface charge (zeta potential value). Representative results of triplicate measures are shown.

Chitosan/pIRES-ARV complexes were synthesized at an N/P ratio of 20 as described in Materials and Methods and as described previously [18]. Under these conditions, the formation of the complexes between pIRES-ARV and CH was confirmed by electrophoretic migration delay in comparison to pIRES-ARV alone, indicating a successful complexation interaction with chitosan (Figure 1(a)). These complexes showed a nanoparticle diameter average close to 100 nm (Figure 1(b)) and a zeta potential value of 1.79 mV (Figure 1(c)). This characterization indicates that complexes between CH and pIRES-ARV (NP-ARV) correspond mainly to nanoparticles of 100 nm of diameter with positive superficial charge.

### 3.2. Expression and Evaluation of the Fusogenic Activity of ARV-p10

**Figure 2:**
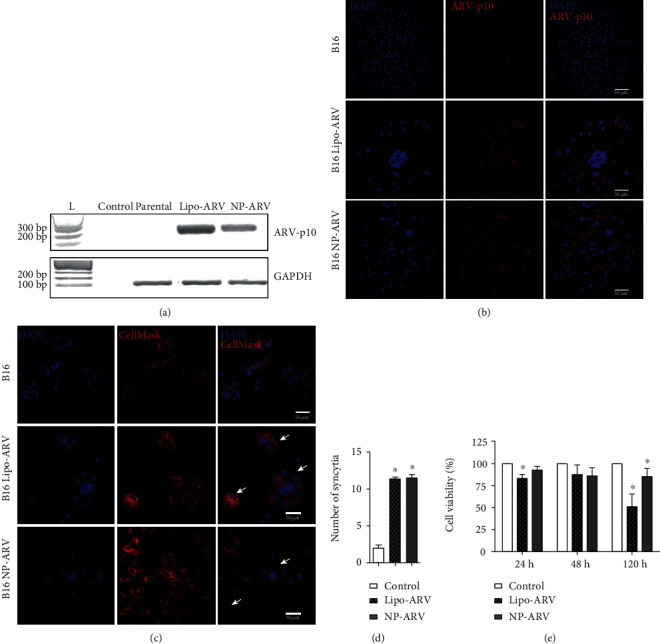
ARV-p10 protein expression in B16 melanoma cells using NP-ARV. (a) The expression of the ARV-p10 protein (upper panel) and housekeeping GAPDH (lower panel) transcripts was determined by RT-PCR 48 hours posttransfection. A representative gel containing DNA ladder (L, lane 1), PCR blank control (control, lane 2), nontransfected cells (parental, lane 3), Lipofectamine pIRES-ARV-transfected cells (Lipo-ARV, lane 4), and NP-ARV-transfected cells (lane 5). (b) Expression of ARV-p10 protein determined by immunofluorescence. B16 (upper panel), Lipo-ARV-transfected B16 cells (middle panel), and NP-ARV-transfected B16 cells (lower panel) stained with DAPI (left column) and with an antibody against ARV-p10 protein (middle column). Merge is shown in the right column. (c) Syncytium formation 48 h posttransfection of B16 cells (upper panel), Lipo-ARV-transfected B16 cells (middle panel), and NP-ARV-transfected B16 cells (lower panel) stained with DAPI (left column) and CellMask (middle column). Merge is shown in the right column. White arrows mark fusion points. (d) Quantification of syncytia 48 hours posttransfection by hemacolor stain. (e) Cell viability was evaluated at 24, 48, and 120 hours posttransfection and was normalized against nontransfected cells. Graphs correspond to average ± standard error of three independent experiments. Statistical analyses were performed using the Mann-Whitney test (^∗^*p* < 0.05).

The ARV-p10 transcript expression and protein expression were evaluated in B16 cells 48 h posttransfection with Lipofectamine/pIRES-ARV (Lipo-ARV) and NP-ARV by RT-PCR and immunofluorescence, respectively. With both transfection methods, the expected amplicon and ARV-p10 protein were detected, but not in nontransfected cells (Figures 2(a) and 2(b)). These results indicate that expression vectors present in the NPs were incorporated by the B16 cells, which allows the expression of the *arv*-*p10* gene and later the ARV-p10 protein synthesis.

The fusogenic activity of ARV-p10 protein was determined by evaluating the presence of syncytia 48 h posttransfection of B16 cells. We observed large multinucleated cells that indicate a syncytium formation process in Lipo-ARV- and NP-ARV-transfected cells, but not in nontransfected cells (Figure 2(c)). The number of formed syncytia in Lipo-ARV and NP-ARV was significantly increased in comparison to nontransfected cells but with no difference between both transfection methods (Figure 2(d)). Moreover, in NP-ARV-transfected cells, the syncytium presence was associated with a 20% decrease in B16 cell viability at 120 hours posttransfection (Figure 2(e)). These results show that the transfection of B16 tumor cells using NP-ARV allows the expression of a fusogenically active ARV-p10 protein, which decreases cell viability because of syncytium formation.

### 3.3. Intratumoral Expression of ARV-p10 Protein with Chitosan Nanoparticles

**Figure 3:**
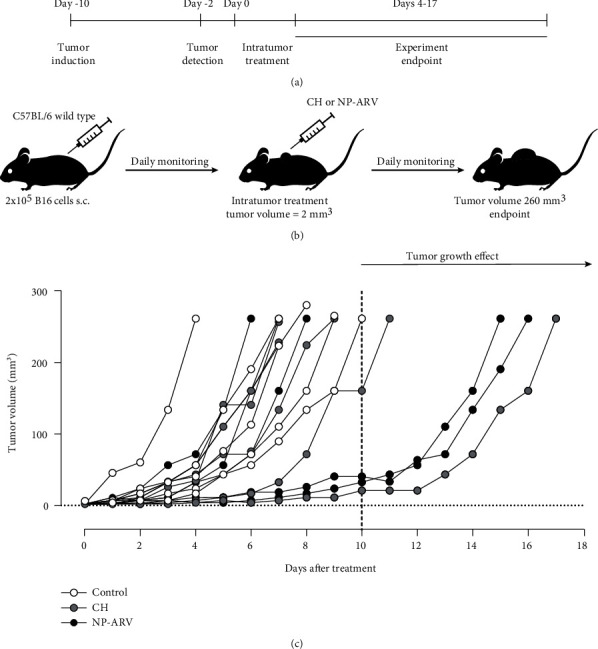
*In vivo* antitumor effect of NP-ARV against melanoma. Mice were challenged with viable B16 cells and monitored daily until tumor size reached its maximum. (a) Experiment timeline. (b) Schematic representation of the experiment strategy. (c) Tumor growth of the nontreated control group (open circles), chitosan alone group (gray circles), and NP-ARV group (black circles). The vertical line at day 10 indicates the day at which the last control mouse reached the MTV. The experiment was done in 5-6 animals for each group and graphed individually.

**Figure 4:**
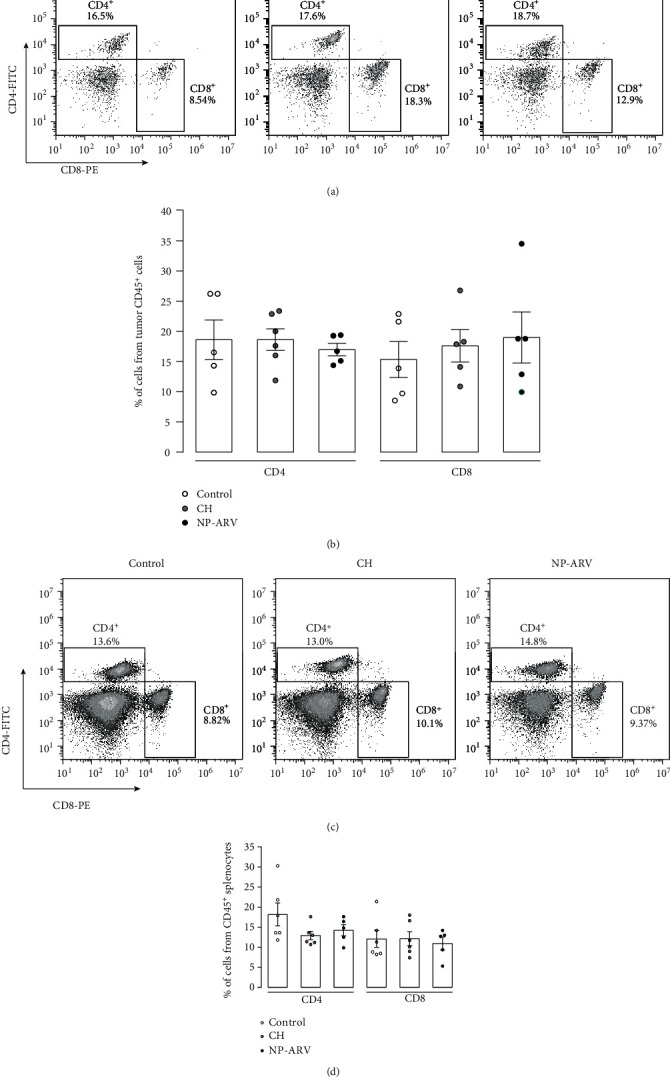
Effect of NP-ARV treatment on tumor-infiltrated and splenic T cells. (a) Representative dot plots of the intratumoral T CD8^+^ and T CD4^+^ lymphocytes are shown for the nontreated animals (control), chitosan alone (CH), and NP-ARV-treated group. (b) Graph bar of the percentage of each population in tumor CD45^+^ cells. (c) Representative dot plots of the splenic T CD8^+^ and T CD4^+^ lymphocytes for the nontreated animals (control), chitosan alone (CH), and NP-ARV-treated group. (d) Graph bar of the percentage of each population in splenic CD45^+^ cells. Bars correspond to average ± standard error; individual experiments are also graphed; statistical analyses were performed using the Mann-Whitney test.

**Figure 5:**
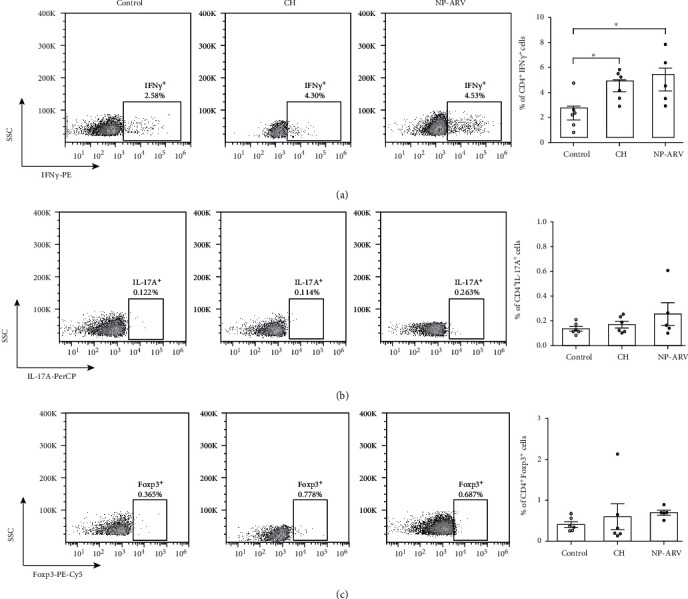
Effect of NP-ARV treatment on splenic CD4^+^ subpopulations. Representative dot plots of splenic T CD4^+^ subpopulation (left) and graph bar of the percentage of each population (right) for the (a) CD4^+^ IFN*γ*^+^, (b) CD4^+^ IL-17A^+^, and (c) CD4^+^ Foxp3^+^ lymphocytes in the nontreated animals (control), chitosan alone (CH), and NP-ARV-treated group. Bars correspond to average ± standard error; individual experiments are also graphed; statistical analyses were performed using the Mann-Whitney test.

Our next aim was to determine whether intratumor (i.t.) injection of NP-ARV would delay the growth of B16 melanoma tumors *in vivo*. For this purpose, mice were challenged with viable B16 cells, reaching a detectable tumor between 6 and 9 days postchallenge. CH nanoparticles alone and NP-ARV were injected i.t. when the tumor reached a volume close to 2 mm^3^ (Figure 3(a)). Animals were monitored daily, evaluating tumor growth until MTV was achieved (Figure 3(b)). The NP-ARV and CH treatments delayed tumor growth on 50% and 20% of mice, respectively, in comparison to control counterparts, but with a nonsignificant difference between both treatments (Figure 3(b)). Despite this tumor growth delay, the CD4^+^ and CD8^+^ lymphocytes in the tumor (Figures 4(a) and 4(b)) and spleen tissue (Figures 4(c) and 4(d)) did not show changes for both treated groups. However, a significant increase in splenic CD4^+^ IFN*γ*^+^ (Th1 lymphocytes) cells was observed in both cases in comparison to the control group (Figure 5(a)). Altogether, these results suggest that the tumor growth delay induced by the CH and NP-ARV treatments could be associated with a Th1 IFN*γ* type of immune response.

## 4. Discussion

Intratumoral expression of viral fusion proteins is a promising strategy as gene therapy against cancer. In this work, we evaluated the effect of *in vitro* and *in vivo* expression of avian reovirus p10 protein (ARV-p10), using chitosan nanoparticles (CH-NPs) as a transfection vehicle of murine B16 melanoma cells. We observed that ARV-p10 expression resulted in syncytium formation associated with a cytotoxic effect *in vitro*, and intratumor treatment with NP-ARV caused a mild delay in tumor development associated with an increase in splenic Th1^+^ IFN*γ*^+^ lymphocytes.

The syncytium generation in B16 tumor cells 48 hours posttransfection with NP-ARV supports the high fusogenic activity described for ARV-p10 protein, classified as a promiscuous fusogen, which is sufficient to induce cell fusion and syncytium formation in various cell lines [7, 19]. Altogether, our results resemble previous studies using a highly fusogenic variant of GALV-F protein, which induces unstable syncytia in human tumor cells, causing potent cytotoxicity *in vitro* [20, 21] and inhibition of tumor growth *in vivo* [20, 22].

Antitumoral reovirus-mediated effects have been associated with changes in immune responses by Prestwich and coworkers, using immunodeficient mice. These authors showed that modifying immune responses is critical for the antitumoral effect of reovirus [23]. Intravenously administered reovirus reduces B16 metastatic lymph nodes and increases antitumor immunity. Similarly, infected Mel888 cells induce DCs and activate autologous peripheral blood lymphocytes [24]; and human DCs loaded with reovirus-infected human melanoma Mel888 cells induce NK cell activation and tumor-specific cytotoxicity [25]. Errington and coworkers demonstrated that reovirus replicates in human melanoma cell lines and i.t. injection of reovirus induces tumor regression in a xenograft model of melanoma, with a mechanism involving modulation of inflammatory responses [26]. In mice, i.t. administration of reovirus into melanoma B16F10 or Lewis Lung Carcinoma (LLC) models prolongs survival and delays tumor growth [27]. In our case, NP-ARV treatment increased cell fusion, delayed tumor growth, and elevated levels of splenic Th1^+^ IFN*γ*^+^ lymphocytes. However, similar results were observed with NP alone, suggesting a possible chitosan NP-mediated effect on Th1 cells. This fact would support previous works showing that chitosan activates dendritic cells [28, 29] and macrophages [30, 31] to release IL-1*β* and IL-12, both cytokines critical for the polarization of CD4 cells to a Th1 phenotype, which is known to participate in antitumor immunity [32].

In conclusion, we showed that the ARV-p10 protein expression using CH-NPs in murine melanoma cells induces efficient tumor cell fusion *in vitro*. This process was associated with a mild cytotoxic effect and an antitumor response *in vivo*. Altogether, these results provide a stepping stone towards future research on improving the ARV-p10 protein expression using CH-NPs as an expression vehicle.

## 5. Conclusion

In conclusion, we proved that *in vitro* ARV-p10 protein expression using CH-NPs in murine melanoma cells induces a cytotoxic effect associated with its cell fusion. However, its use to treat melanoma tumors produces no difference in *in vivo* antitumoral impact in comparison to chitosan treatment. Further studies are necessary for establishing a protocol for efficient *in vivo* DNA delivery of fusion proteins to produce an antitumoral effect.

## Data Availability

The data used to support the findings of this study are available from the corresponding author upon request.
